# A Simple, Low Cost, Sensitive, and Portable Electrochemical Immunochromatography Sensing Device to Measure Estrone-3-Sulfate

**DOI:** 10.3390/s20174781

**Published:** 2020-08-24

**Authors:** Wataru Iwasaki, Chiwa Kataoka, Kazuyuki Sawadaishi, Keitaro Suyama, Nobutomo Morita, Masaya Miyazaki

**Affiliations:** 1 Sensing System Research Center, National Institute of Advanced Industrial Science and Technology, 807-1 Shuku-Machi, Tosu, Saga 841-0052, Japan; morita.nobutomo@aist.go.jp; 2 Carbuncle BioScienTech LLC, 40-26 Tanida, Okukaiinji, Nagaokakyo, Kyoto 617-0853, Japan; cbst_kataoka@mac.com (C.K.); cbst_sawadaishi@mac.com (K.S.); 3 Faculty of Arts and Science, Kyushu University, 744 Motooka, Nishi-ku, Fukuoka 819-0395, Japan; suyama@artsci.kyushu-u.ac.jp; 4 Center of Plasma Nano-Interface Engineering, Kyushu University, 744 Motooka, Nishi-ku, Fukuoka 819-0395, Japan; miyazaki.masaya.833@m.kyushu-u.ac.jp

**Keywords:** POCT, microfluidic paper-based analytical device, electrochemical immunochromatography, estrone-3-sulfate, competitive immunoassay

## Abstract

In livestock production, point-of-care testing (POCT) technology that enables easy on-site analysis of sex hormones is desired to improve reproductive efficiency. In this context, low-molecular-weight endogenous steroids are particularly important for perinatal management. Therefore, we attempted to use a simple method that combines electrochemical techniques with immunochromatography to measure estrone-3-sulfate (E_1_S), one of the low-molecular-weight endogenous steroids that is an estrogen ester. The limit of detection (LOD) for E_1_S achieved by electrochemical immunochromatography was 570.5 ng/mL, which was one to two orders of magnitude lower than that of small molecule compounds analyzed by other POCT techniques (Primpray et al., *Anal. Chim. Acta*, 2019). In addition, it was indicated by a colorimetric analysis that the sensitivity of the electrochemical immunochromatographic technique could be enhanced by improving the method of application of the antibodies on the nitrocellulose membrane and the contact between the electrochemical detector and the nitrocellulose membrane.

## 1. Introduction

Low-molecular-weight steroids, such as estrogen and progesterone, are important sex hormones that are measured to detect ovulation, pregnancy, and predict birth. In cattle production, conception rates have been declining over the past few decades, necessitating an improvement in reproductive efficiency [1,2]. Therefore, techniques to monitor these sex hormones and detect the optimal artificial insemination period, pregnancy, and birth period are required. For example, in the case of wagyu calving, there is a risk of dystocia and death of the calf, and the mother requires the constant assistance of the farmers at the barns during the calving season, thus putting them under a large burden. Estrone-3-sulfate (E_1_S) is expected to be a candidate for estimating the calving period. The level of E_1_S is known to increase in the blood during pregnancy and decrease rapidly just before calving [3]. The levels of other sex hormones are also known to increase in the blood prior to calving; however, it is difficult to set a threshold value on their levels in the blood, which increase because of individual differences. On the other hand, since the blood concentration of E_1_S decreases prior to calving, the time of calving can be predicted by detecting this change.

Usually, radioimmunoassay (RIA) and enzyme-linked immunosorbent assay (ELISA) are used to measure the E_1_S levels in blood [4,5]; however, these methods require skilled labor and a long analysis time. Recently, a simpler electrochemical method for E_1_S analysis was reported [6], in which E_1_S present in a sample was adsorbed to an electrochemical sensor and detected. The sensor was first immersed in a standard solution containing [Fe(CN)_6_]^3−/4−^, followed by a sample solution containing E_1_S; subsequently, the standard solution was measured again. The detection signal was reduced due to E_1_S adsorption to the sensor, which demonstrated a high sensitivity of 1.18 pM (439.4 pg/mL), but this system is a batch method and is not suitable for analysis at the production fields. In recent years, point-of-care testing (POCT) technology has been developed as a method for on-site analysis. In particular, microfluidic paper-based analytical devices (µPADs) are attracting a lot of attention as POCT because they are inexpensive, have easy fabrication and operation, and do not require a pump [7]. Various detection attempts have been made with µPADs [8,9,10,11,12], yet the electrochemical method is considered to be more sensitive than the colorimetric method [13]. On the other hand, µPADs are superior to other methods, such as ELISA, in terms of simplicity, but are inferior in terms of sensitivity. In addition, it is difficult to analyze small molecules, such as steroids, by sandwich ELISA, and the only way to analyze them is by competitive ELISA; therefore, the sensitivity tends to be poor. For example, it was reported that dopamine, which is one of the steroids, was electrochemically measured within the detection range of 10 nM to 1 µM [14]. In contrast, dexamethasone and prednisolone that could not be measured electrochemically were measured by competitive immunoassay and they demonstrated reduced sensitivity with their limit of detection (LOD) at 3.59 µg/mL and 11.98 µg/mL, respectively [15]. In another study, creatinine, which is one of the small molecule compounds, was electrochemically measured in the range from 18.75 µg/mL to 150 µg/mL by measuring the levels of hydrogen peroxidase produced by the enzymatic reaction of creatinine with three enzymes—namely creatininase, creatinase, and sarcosine oxidase [12].

We have previously developed an electrochemical immunochromatography platform as a µPAD [16,17]. Usually, electrodes are printed on a paper when an electrochemical method is introduced into µPADs [9,18,19]. In such a case, the electrodes would be disposed of along with the paper, thus making it costly. In addition, the printed electrode fills the porous membrane and obstructs the flow by capillary force. On the contrary, our device has separate electrodes and membranes, and the electrodes fabricated by gold sputtering have a flat surface that allows sensitive detection. In the present study, we tried to measure E_1_S by competitive immunoassay using this electrochemical immunochromatography platform.

## 2. Materials and Methods

### 2.1. Reagents and Apparatus

A nitrocellulose membrane (HF120, Merck Ltd., Tokyo, Japan) was used as an immunochromatographic strip for the electrochemical immunochromatographic assay. E_1_S sodium salt (E0254) was purchased from Sigma-Aldrich (Tokyo, Japan). Custom-made anti-E_1_S antibody (E3SA4-03-1) and alkaline phosphatase labeled E_1_S (E_1_S-ALP, 20-221D-6) were purchased from Carbuncle BioScienTech LLC (Kyoto, Japan). Substrates for the electrochemical and colorimetric methods, viz. *p*-aminophenylphosphate monosodium salt (*p*APP, A5030) and *p*-nitrophenylphosphate (*p*NPP, 37621), respectively, were purchased from LTK Laboratories, Inc. (St Paul, MN, USA) and Thermo Fisher Scientific K.K. (Tokyo, Japan), respectively. 5-bromo-4-chloro-3-indolyl phosphate/nitro blue tetrazolium chloride (BCIP/NBT, B5655) purchased from Sigma-Aldrich (Tokyo, Japan) was used as a colorimetric substrate for immunochromatography. Block ACE (UKB 40) purchased from MEGMILK SNOW BRAND Co., Ltd. (Tokyo, Japan) and SuperBlock^TM^ T20 (TBS). Blocking Buffer purchased from Thermo Fisher Scientific K.K. (Tokyo, Japan) was used as a blocking buffer for ELISA and electrochemical immunochromatography, respectively. Ultrapure water was prepared with Direct-Q UV3 from Merck Ltd. (Tokyo, Japan). An electrochemical analyzer (ALS630D) and Ag/AgCl ink for the reference electrode were purchased from BAS Inc. (Tokyo, Japan). A microplate reader (iMark^TM^) was purchased from Bio-Rad Laboratories, Inc. (Berkeley, CA, USA).

### 2.2. Electrochemical Immunochromatography Platform

**Figure 1:**
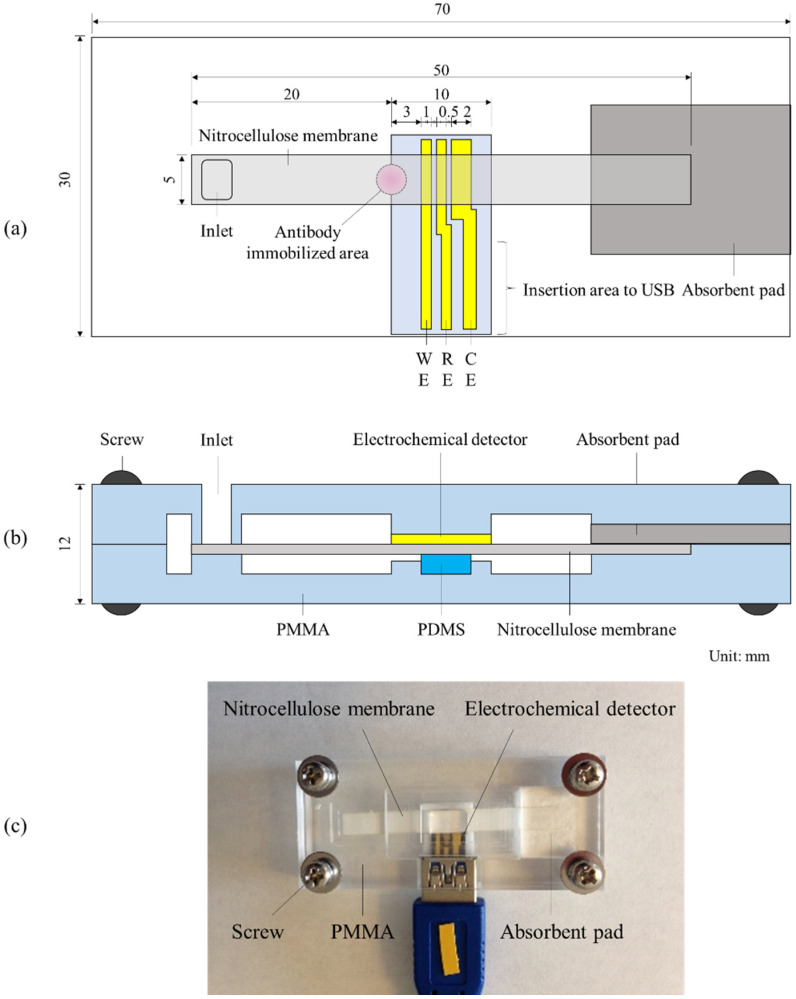
Electrochemical immunochromatography platform. (**a**) The layout of the nitrocellulose membrane and electrochemical detector. (**b**) Schematic of cross-sectional view. (**c**) Photograph of top view [17]. WE, RE, and CE represent the working electrode, reference electrode, and counter electrode, respectively. The unit of the value is millimeters. The platform consists of a nitrocellulose membrane, an absorbent pad, an electrochemical detector, and two polymethyl methacrylate (PMMA) plates. The electrochemical detector and the absorbent pad were attached to the nitrocellulose membrane at the center and at the edge, respectively. The structure was designed to stabilize the contact state mediated by tightening with controlled torque and pressing with polydimethylsiloxane (PDMS). The top PMMA plate had an inlet at the upstream of the nitrocellulose membrane.

The electrochemical immunochromatography platform, which we previously developed [17], was used for the electrochemical detection of E_1_S. The platform consists of a nitrocellulose membrane, an absorbent pad, an electrochemical detector, and two polymethyl methacrylate (PMMA) plates (Figure 1). The electrochemical detector was fabricated by standard photolithography and the liftoff process, which has been previously described [16]. The photoresist was briefly coated on a silicon wafer with a 1 µm thick thermal oxide film. The electrode pattern was exposed on the wafer, and the photoresist of the exposed area was removed by developing it. Furthermore, Cr (10 nm) and Au (200 nm) were deposited by EB deposition, and the remaining photoresist was removed. The gold electrode pattern was thus fabricated on the silicon wafer. The silicon wafer was divided into 10 mm × 20 mm sections, and Ag/AgCl ink was coated on the reference electrode and dried at 70 °C for 2 h, thus resulting in the electrochemical detector.

A nitrocellulose membrane with a flow rate of 120 s/ 4 cm, generally used for immunochromatography, was cut into a 5 mm wide and 50 mm long piece. The electrochemical detector and the absorbent pad were attached to the nitrocellulose membrane at the center and the edge, respectively. The antibody immobilized area on the nitrocellulose membrane was located 20 mm from the top edge of the membrane and its center was 3 mm upstream of the working electrode. This was done to allow for the measurement of all the electrochemical species produced in the antibody immobilized area (Figure 1a). In addition, the structure was designed to stabilize the contact state mediated by tightening with controlled torque and pressing with polydimethylsiloxane (PDMS) (Figure 1b), because the measurement current is proportional to the area of contact between the electrode and the liquid. The screw was tightened with a constant, weak torque to avoid the possibility of crushing the membrane and making the flow unstable resulting from the excessive pressure applied. The liquid on the surface of the nitrocellulose membrane was considered to be in contact with the electrode at the stable contact area (1 mm × 5 mm). The top PMMA plate had an inlet at the upstream of the nitrocellulose membrane.

### 2.3. Enzyme-Linked Immunosorbent Assay

We performed ELISA to evaluate the custom-made anti-E_1_S antibody and E_1_S-ALP. Standard E_1_S solutions were prepared by dissolving E_1_S sodium salt in ultrapure water to obtain various concentrations (0.003, 0.01, 0.03, 0.1, 0.3, 1, 3, 10, and 30 ng/mL). At first, 50 µL of primary antibody solution (PBS containing 0.5 µg/mL of anti-E_1_S antibody) was added to the wells of a microplate and incubated for 1 h. Next, the anti-E_1_S antibody solution was discarded from each well and the wells were washed with PBS containing 0.05% Tween20 (PBST) three times. Next, 250 µL of blocking buffer (4x diluted block ACE) was added to the wells and incubated for 2 h. Each well was washed again thrice with PBST. Furthermore, 25 µL of standard E_1_S solution and 25 µL of E_1_S-ALP solution (150 mM MgCl_2_ containing 0.03 µg/mL of E_1_S-ALP) were added to each well and incubated for 1 h. Subsequently, the wells were washed with PBST thrice, and 50 µL of *p*NPP solution was added to each well and incubated for 30 min. Finally, 50 µL of 2 M NaOH was added to stop the enzyme reaction of ALP and *p*NPP, and the absorbance was measured at 405 nm using a microplate reader.

### 2.4. Electrochemical Immunochromatography

**Figure 2:**
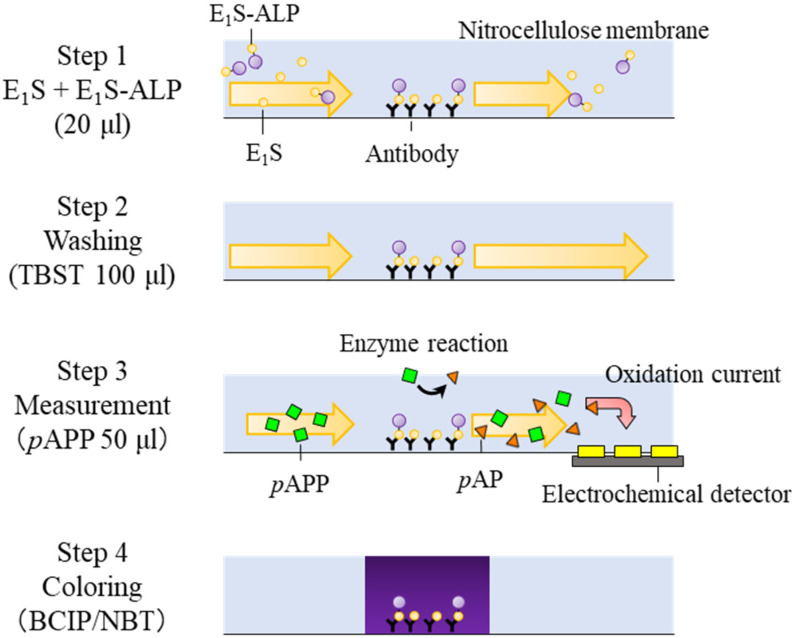
Measurement procedure of competitive immunoassay in the electrochemical immunochromatography. Firstly, 10 µL of E_1_S solution and 10 µL of E_1_S-ALP solution (50 ng/mL of E_1_S-ALP in 20 mM tris buffered saline containing 0.05% Tween20 (TBST) containing 0.1% bovine serum albumin (BSA)) were added to the inlet of the electrochemical immunochromatography platform. E_1_S and E_1_S labeled with alkaline phosphatase (E_1_S-ALP) competed with each other and were captured by the immobilized anti-E_1_S antibody. Secondly, 50 µL of 20 mM TBST was added twice to flush out the untrapped E_1_S and E_1_S-ALP. Thirdly, 50 µL of 20 mM TBST containing 1 mg/mL of *p*APP and 50 mM KCl were added to the inlet. The *p*APP reacted with the alkaline phosphatase of E_1_S-ALP captured by the immobilized anti-E_1_S antibody and produced *p*-aminophenol (*p*AP) that was run down the membrane and detected by the electrochemical detector. Finally, the nitrocellulose membranes were colored by immersing in 5-bromo-4-chloro-3-indolyl phosphate/nitro blue tetrazolium chloride (BCIP/NBT) solution for 15 min and washed with ultrapure water for 10 min to check the condition of the anti-E_1_S antibodies and E_1_S-ALPs.

We prepared an immunochromatographic strip before the electrochemical immunochromatography measurements were carried out. Anti-E_1_S antibody was immobilized on an immunochromatographic strip by dropping 2 µL of anti-E_1_S antibody solution (5 mM phosphate buffer pH 7.3 containing 1 mg/mL of primary antibody) on a nitrocellulose membrane at 20 mm from the upstream edge as shown in Figure 1a, and then the membrane was incubated at 37 °C for 2 h. The membrane was immersed in the blocking buffer and maintained in a shaking condition for 15 min. Next, it was washed by immersion in 20 mM tris buffered saline (TBS) containing 0.05% Tween20 (TBST) and incubated in shaking condition for 30 min. The membrane was dried at 4 °C overnight. E_1_S measurement by electrochemical immunochromatography was performed as follows (Figure 2). At first, 10 µL of E_1_S solution and 10 µL of E_1_S-ALP solution (50 ng/mL of E_1_S-ALP in 20 mM TBST containing 0.1% bovine serum albumin (BSA)) were added to the inlet of the electrochemical immunochromatography platform. The concentrations of E_1_S were 1, 10, 100, 1000, and 10000 ng/mL. E_1_S and E_1_S-ALP solutions flowed downstream through the membrane due to capillary force. E_1_S and E_1_S-ALP competed with each other and were captured by the immobilized anti-E_1_S antibody. Untrapped E_1_S and E_1_S-ALP were flushed out by running 50 µL of 20 mM TBST twice. Thereafter, 50 µL of 20 mM TBST containing 1 mg/mL of *p*APP and 50 mM KCl were added to the inlet. The *p*APP reacted with the alkaline phosphatase of E_1_S-ALP captured by the immobilized anti-E_1_S antibody and produced *p*-aminophenol (*p*AP) that was run down the membrane and detected at the electrochemical detector. We conducted each measurement 3 times. After obtaining the electrochemical measurements, the nitrocellulose membranes used for electrochemical immunochromatography were colored by immersing in BCIP/NBT solution for 15 min and washed with ultrapure water for 10 min to check the condition of the anti-E_1_S antibodies and E_1_S-ALPs.

## 3. Results and Discussion

### 3.1. ELISA

**Figure 3:**
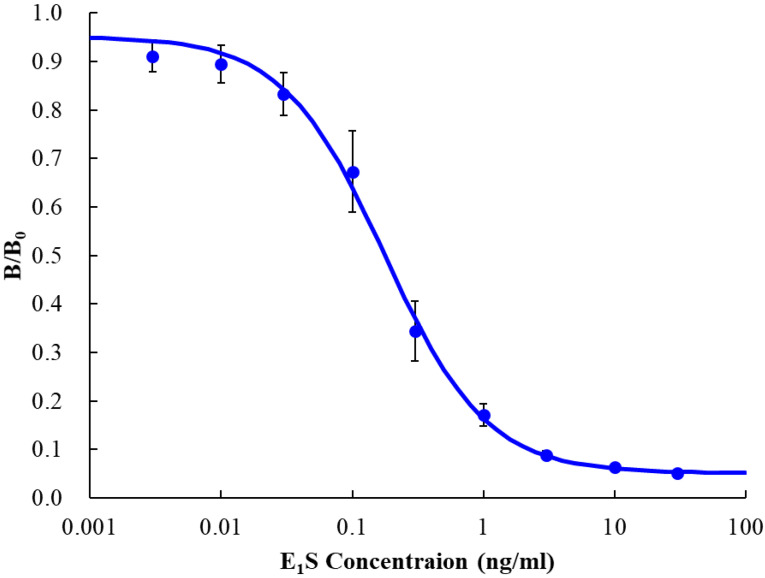
Calibration curve obtained by ELISA (n = 6). The vertical axis of the graph shows B/B_0_, where B and B_0_ are the absorbances of each sample and blank, respectively. The error bars represent standard deviations.

We performed ELISA to evaluate the performance of the anti-E_1_S antibody and E_1_S-ALP. We succeeded in obtaining a calibration curve with a small variation (Figure 3). The vertical axis of the graph shows B/B_0_, where B and B_0_ are the absorbance of each sample and blank, respectively. The absorbance of the blank was 0.608 ± 0.015 and the LOD was 7.8 pg/mL (blank + 3σ). It is considered that the LOD of this ELISA test was sufficient and that the anti-E_1_S antibody and E_1_S-ALP had performed sufficiently, since the nominal LOD of the commercialized ELISA kit for E_1_S (EIA17E3S, Thermo Fisher Scientific), which is a horseradish peroxidase enzyme reaction system, is 26.4 pg/mL.

### 3.2. Electrochemical Immunochromatography Measurement

**Figure 4:**
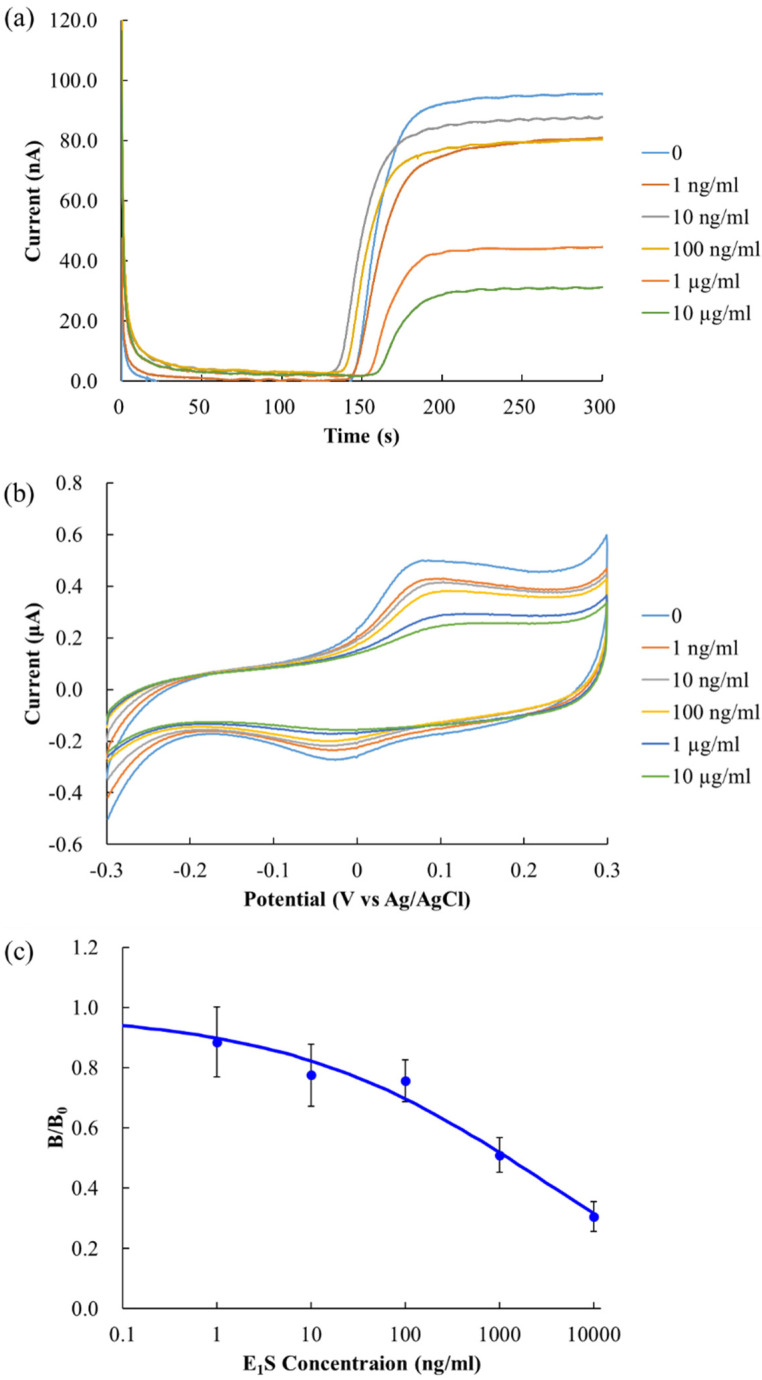
Results of electrochemical immunochromatography for E_1_S. (**a**) Chronoamperogram at the applied potential of 0.15 V vs Ag/AgCl. (**b**) Cyclic voltammogram at the incremental potential of 1 mV and scan rate of 50 mV/s. (**c**) Calibration curve (n = 3). The vertical axis of the graph shows B/B_0_, where B and B_0_ are the electrochemical currents at 250 s of chronoamperogram for each sample and blank, respectively. Error bars represent standard deviations.

Figure 4a shows a chronoamperogram of each concentration of E_1_S obtained through electrochemical immunochromatography. We started chronoamperometry just after *p*APP was added to the inlet. The applied potential was 0.15 V vs. Ag/AgCl. The electrochemical signal rapidly increased around 150 s, because *p*AP reached the electrochemical detector, after which a stable signal was observed. Cyclic voltammetry was performed after 300 s of chronoamperometry under the following conditions: potential range = −0.3 V to 0.3 V, incremental potential = 1 mV, scan rate = 50 mV/s. The cyclic voltammogram is shown in Figure 4b. The potential at which the peak of the *p*AP oxidation current was observed was dependent on the concentration of E_1_S, i.e., on the concentration of *p*AP, and was observed at a potential of 0.07 V to 0.12 V.

The oxidation current at 250 s of chronoamperometry for each concentration was divided with that of the blank. The values (B/B_0_) were used for the calibration curve (Figure 4c). Each point in the plot was an average of triplicate measurements. The blank value was 97.5 ± 14.1 nA, and the LOD (blank + 3σ) was 570.5 ng/mL. The results demonstrated LODs that were one to two orders of magnitude lower than those reported in other studies that analyzed small molecule steroids, namely dexamethasone and prednisolone, by electrochemical analysis of paper analysis chips [15]. Conversely, although the electrochemical signal showed a decreasing change over the entire range, the LOD was poorer than that of ELISA due to the high variability of the signal, with a variance of 9–16% at each concentration. It is well known that the electrochemical signal is affected by the flow velocity in the direction parallel to the plate electrode [17,20], and the velocity variance of the nitrocellulose membrane used is nominal—about 8%, according to the manufacturer. It is also possible that the variability in flow velocity affects the concentration of the reactants per unit time and the reaction time of the antigen–antibody and enzymatic reactions.

### 3.3. Colorimetric Evaluation of Electrochemical Immunochromatography Measurement

**Figure 5:**
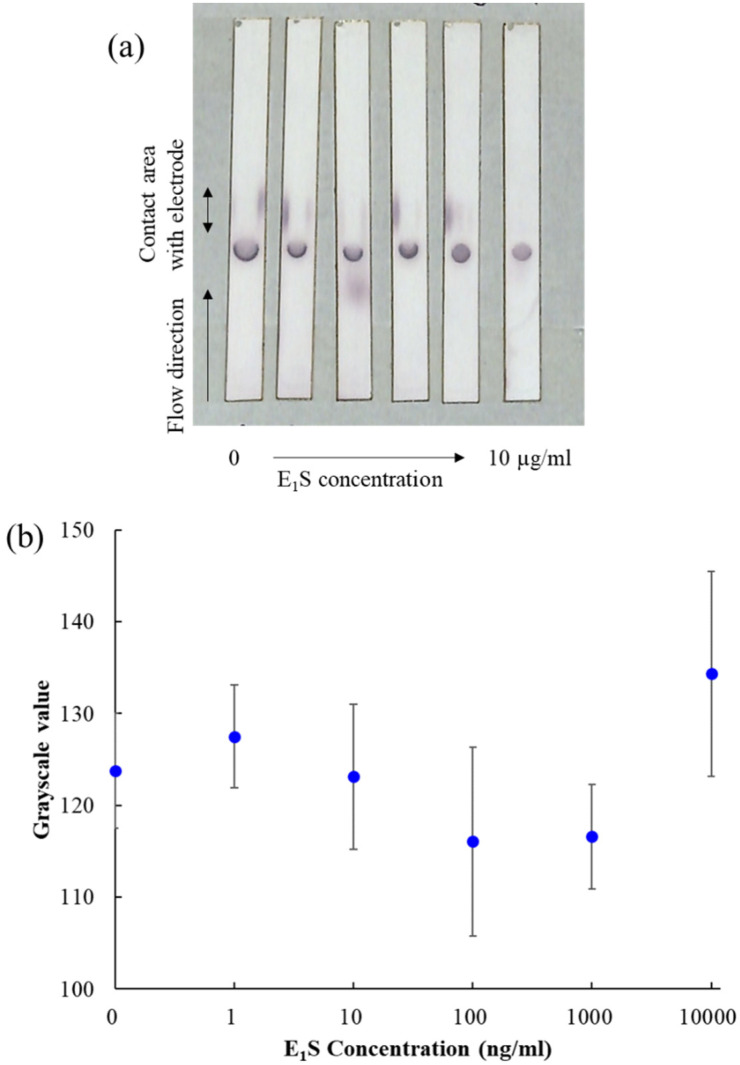
Results of colorimetric method. (**a**) Photograph of a colored nitrocellulose membrane after being used for electrochemical immunochromatography. (**b**) The average 8-bit grayscale value for the antibody-immobilized area at each E_1_S concentration (n = 3). The grayscale value was calculated using Adobe Photoshop.

Photographs of the nitrocellulose membrane after electrochemical measurement are shown in Figure 5a. Upon colorimetric evaluation, the antibody-immobilized area appeared colored, indicating that E_1_S-ALP was efficiently captured by the immobilized anti-E_1_S antibody. The intensity of the coloration of the antibody immobilized area was quantified by Adobe Photoshop (Adobe KK, Tokyo, Japan), as well as a previously reported technique [21]. The color photograph was converted to 8-bit grayscale, and the average grayscale value of the antibody-immobilized area was calculated. The average triplicate measurement of each E_1_S concentration is shown in Figure 5b. No relationship was observed between the grayscale value and the E_1_S concentration. The colorimetric measurement was performed after the electrochemical measurement, i.e., after the enzymatic reaction was performed, which did not necessarily yield accurate results, but suggested that the electrochemical measurements were more sensitive than the colorimetric measurements. On the other hand, the areas other than those specific to antibody immobilization were also colored, indicating the presence of alkaline phosphatase in these areas as well. Since the electrochemical signal measured by the electrochemical detector reflects the total amount of *p*AP produced in the reaction with ALP both inside and outside the antibody immobilization area, it is likely to affect the variability of the measured signal. Therefore, the blocking step needs to be made more stringent. The downstream side of the antibody immobilization section was colored, especially in the areas at both ends that are in contact with the electrochemical detector. This area is essentially pressed upon with light force to improve contact conditions between the electrochemical detector and the nitrocellulose membrane, which could have reduced the flow and prevented the E_1_S-ALP from flowing downstream during washing. This E_1_S-ALP may have resulted in the variability of the detection signal. Since the area just behind the antibody immobilization zone was almost colorless, it is assumed that most of the E_1_S-ALP passing through the immobilization zone was captured by the antibody, and that which did not pass through the antibody immobilization zone is thought to have remained at the electrode. Therefore, this problem could be solved by spreading the antibody across the entire width of the nitrocellulose membrane, as is done in conventional immunochromatography, to reduce the variability of the measurement signal.

## 4. Conclusions

We measured the small molecule steroid E_1_S by competitive immunoassay using our previously developed electrochemical immunochromatography platform. Electrochemical immunochromatography demonstrated an LOD of 570.5 ng/mL, which was one to two orders of magnitude better than that of other studies that analyzed small molecule steroids, namely dexamethasone and prednisolone, by electrochemical analysis of paper analysis chips [15]. In addition, the electrochemical method was shown to be superior to the colorimetric method in immunochromatography. However, the LOD of electrochemical immunochromatography was less sensitive than ELISA, which could be attributed to the large variability in the signal of electrochemical immunochromatography. Variations in the flow velocity and non-specific adsorption of ALP outside the antibody application region were suggested to be responsible for the variation in the signal of electrochemical immunochromatography. The flow rate variability in the nitrocellulose membranes is a common issue with normal immunochromatography, and higher quality nitrocellulose membranes need to be prepared to counter this. Some practical ways of improving the variability in the signal include optimizing the blocking and antibody application methods and re-examining the contact method between the electrode and the nitrocellulose membrane. By solving these issues, improvement in the sensitivity of electrochemical immunochromatography may be achieved. In this study, we used a desktop PC and a desktop potentiostat for the electrochemical immunochromatography measurements; however, it is possible to construct a portable system by using a USB-type potentiostat and a tablet PC available in the market. In the future, solving these issues will allow for a more sensitive analysis of small molecule analytes by POCT.
